# Longer Cortical Silent Period Length Is Associated to Binge Eating Disorder: An Exploratory Study

**DOI:** 10.3389/fpsyt.2020.559966

**Published:** 2020-10-14

**Authors:** Luciana C. Antunes, Jessica Lorenzzi Elkfury, Cristiane Schultz Parizotti, Aline Patrícia Brietzke, Janete Shatkoski Bandeira, Iraci Lucena da Silva Torres, Felipe Fregni, Wolnei Caumo

**Affiliations:** ^1^Associate Professor in the Health Science Center, Nutrition Department, Universidade Federal de Santa Catarina (UFSC), Florianópolis, Brazil; ^2^Post-Graduate Program in Medical Sciences, School of Medicine, Universidade Federal do Rio Grande do Sul (UFRGS), Porto Alegre, Brazil; ^3^Associate Professor, Pharmacology Department, Instituto de Ciências Básicas da Saúde, Universidade Federal do Rio Grande do Sul (UFRGS), Porto Alegre, Brazil; ^4^Neuromodulation Center and Center for Clinical Research Learning, Spaulding Rehabilitation Hospital and Massachusetts General Hospital, Boston, MA, United States; ^5^Anesthesiologist, Pain and Palliative Care Service at Hospital de Clínicas de Porto Alegre (HCPA), Laboratory of Pain and Neuromodulation at Universidade Federal do Rio Grande do Sul (UFRGS), Porto Alegre, Brazil; ^6^Associate Professor of Pain and Anesthesia, Surgery Department, School of Medicine, Universidade Federal do Rio Grande do Sul (UFRGS), Porto Alegre, Brazil

**Keywords:** binge-eating disorder, obesity, cortical excitability, eating behavior, executive function, eating disorders

## Abstract

**Introduction:** Although binge eating disorder (BED) is an eating disorder and obesity is a clinical disease, it is known that both conditions present overlapped symptoms related to, at least partially, the disruption of homeostatic and hedonistic eating behavior pathways. Therefore, the understanding of neural substrates, such as the motor cortex excitability assessed by transcranial magnetic stimulation (TMS), might provide new insights into the pathophysiology of BED and obesity.

**Objectives:** (i) To compare, among BED, obesity, ex-obese, and HC (healthy control) subjects, the cortical excitability indexed by TMS measures, such as CSP (cortical silent period; primary outcome), SICI (intracortical inhibition), and ICF (intracortical facilitation; secondary outcome). (ii) To explore the relationship of the CSP, eating behavior (e.g., restraint, disinhibition, and hunger), depressive symptoms, and sleep quality among the four groups (BED, obesity, ex-obese, and HC).

**Methods:** Fifty-nine women [BED (*n* = 13), obese (*n* = 20), ex-obese (*n* = 12), and HC (*n* = 14)] comprise the total sample for this study. Assessments: cortical excitability measures (CSP, SICI, and ICF), inhibition response task by the Go/No-go paradigm, and instruments to assess the eating psychopathology (Three-Factor Eating Questionnaire, Eating Disorder Examination Questionnaire, and Binge Eating Scale) were used.

**Results:** A MANCOVA analysis revealed that the mean of CSP was longer in the BED group compared with other three groups: 24.10% longer than the obesity group, 25.98% longer than the HC group, and 25.41% longer than the ex-obese group. Pearson's correlations evidenced that CSP was positively associated with both eating concern and binge eating scores.

**Conclusion:** The findings point out that BED patients present longer CSP, which might suggest an upregulation of intracortical inhibition. Additionally, CSP was positively correlated with Binge Eating Scale and eating concern scores. Further studies are needed.

## Introduction

Binge eating disorder (BED) is characterized by the consumption of large amounts of food associated with an experienced loss of control (1). Although BED is a psychiatric disorder and obesity is a medical condition, both present overlapped symptoms related to, at least somehow, the disruption of homeostatic and hedonistic eating behavior pathways (2). According to studies that used behavioral, neurobiological, and neuroimage techniques, the overeating habit is associated with top-down psychological processes, such as disinhibition, impulsivity, and risk-taking propensity (3). Obese individuals with BED show increased impulsivity (rash-spontaneous behavior) compared to obese without BED and normal-weight individuals, in general or in food-specific tasks that can denote a distinct phenotype within the obesity spectrum (4). Corroborating these findings, neuroimage data revealed that BED and obesity showed some abnormal patterns in the frontal region of the brain, as well as the mesocorticolimbic circuits involved in reward processing and decision-making (5, 6). These dysfunctions indicate that neuroplastic networks mediate the equipoise in reward neural networks, and the homeostatic system stands out as a possible shared mechanism between these two conditions (7). Studies performed in BED and/or in obese subjects found impaired executive control compared to healthy controls (HC) (8). BED patients have displayed stronger inhibitory deficits on the Go/No-go task compared with obese individuals (4, 9). On the other hand, in both BED (10) and obesity (11), a diminished ability to inhibit responses related to food on the Go/No-go task compared with lean controls has been evidenced. These data suggest that the poor decision-making and the uncontrolled impulse may be facilitated, having as a consequence overeating in both groups.

Impulse control involves several neurobiological systems, such as dopaminergic, gamma-aminobutyric acid (GABA-ergic), glutamatergic, serotonergic, etc. (12). Whereas the neuronal circuits involved in the hedonic order are in the corticolimbic order, and their signaling methods include dopaminergic and opioid pathways (12), in this circuitry, the GABAergic interneurons contribute to the disinhibitory control of reward-related response (12). In subcortical regions, the basal ganglia, such as the nucleus accumbens (NAc) has GABA-containing cells, with projections to thalamus, midbrain, and brainstem (12). Thereby, inhibitory circuits can lead to maladaptive behavioral changes, such as overeating (13). In particular, inhibitory synaptic plasticity within the mesocorticolimbic system alters circuit function and mediates behavioral adaptations related to stress (13). In this pathway, the dopaminergic neurons received GABAergic inputs from other neural networks and GABA inhibits mesolimbic dopamine signaling in the ventral tegmental area (VTA) (12). Thus, the dysfunction in the inhibitory neural networks emerges as a possible mechanism underlying eating disorders and comprises the rationale to use neuromodulatory techniques as a therapeutic option [e.g., transcranial magnetic stimulation (TMS)] as recently proposed by Stramba-Badiale et al. (6).

Neuromodulatory techniques, such as TMS, have been utilized not only as a diagnostic tool to access cortical excitability of a target brain region, being an indirect measure of the activity of GABA receptors, but also as treatment strategies [repetitive TMS (rTMS)] for a variety of neurologic and psychiatric disorders. Administration of single or multiple sessions of rTMS over right and/or left dorsolateral prefrontal cortex (DLPFC) showed reduction in craving, weight, and eating disorder (ED) symptoms (6, 14, 15).

Thereby, it is reasonable to consider the motor cortex excitability measured by TMS as probing neural plasticity indexes to improve the comprehension of the neural substrates shared by BED and obesity, as well as their interplay with measures of impulsivity, and with impulse control by behavioral paradigms, such as the Go/No-go. Thus, this study tested the hypothesis that BED patients would present higher disinhibition of the motor cortex compared to obese, ex-obese, and HC subjects. The aims of this study were the following: (i) To compare, among subjects with BED, obesity, ex-obese, and HC, the cortical excitability indexed by TMS measures, such as CSP (cortical silent period; primary outcome), SICI (intracortical inhibition), and ICF (intracortical facilitation; secondary outcome). (ii) To explore the relationship of the CSP, eating behavior (e.g., restraint, disinhibition, and hunger), depressive symptoms, and sleep quality among the four groups (BED, obesity, ex-obese, and HC).

## Methods and Materials

### Study Design, Settings, and Subjects

The researchers conducted a cross-sectional study following the Strengthening the Reporting of Observational Studies in Epidemiology (STROBE) statement. The Ethics Committee Board of the Hospital de Clínicas de Porto Alegre (HCPA) (Institutional Review Board IRB 70321817900005327) approved the protocol. All subjects provided oral and written consent before their engagement in the study.

### Participants

Volunteers were outpatients from primary health care units or psychiatric outpatient clinics and tertiary hospitals (obese and BED groups, respectively), enlisted from the local community by advertisement postings in universities and public places in Porto Alegre, Brazil. The sample included literate, female, self-reported right-handed, 18- to 65-year-old subjects. The common exclusion criteria were formal contraindication for TMS (e.g., pregnancy, history or diagnostic of seizure, metal implants in the head, etc.), history of alcohol abuse or other drugs in the past 6 months, individuals undergoing weight loss treatment, bariatric surgery individuals, and night shift workers.

Obese and lean individuals were diagnosed according to World Health Organization (WHO) criteria (BMI > 29.99 kg/m^2^ and between 18.5 and 24.99 kg/m^2^, respectively). Volunteers classified as lean, according to BMI, at the time of the interviews and who had a clinical history of obesity (BMI > 29.99 kg/m^2^) at least 6 months before the study, according to medical or nutritional records, were assigned to the Ex-obese group.

Diagnosis of BED was performed according to the diagnostic criteria outlined in the Diagnostic And Statistical Manual Of Mental Disorders, 5th Edition (1), applied by a trained mental health professional. Lean individuals (HC and Ex-obese group) were excluded if any eating disorder diagnosis were confirmed. Obese individuals were included in the BED group if BED diagnosis was confirmed and excluded if any other eating disorder was diagnosed.

**Figure 1:**
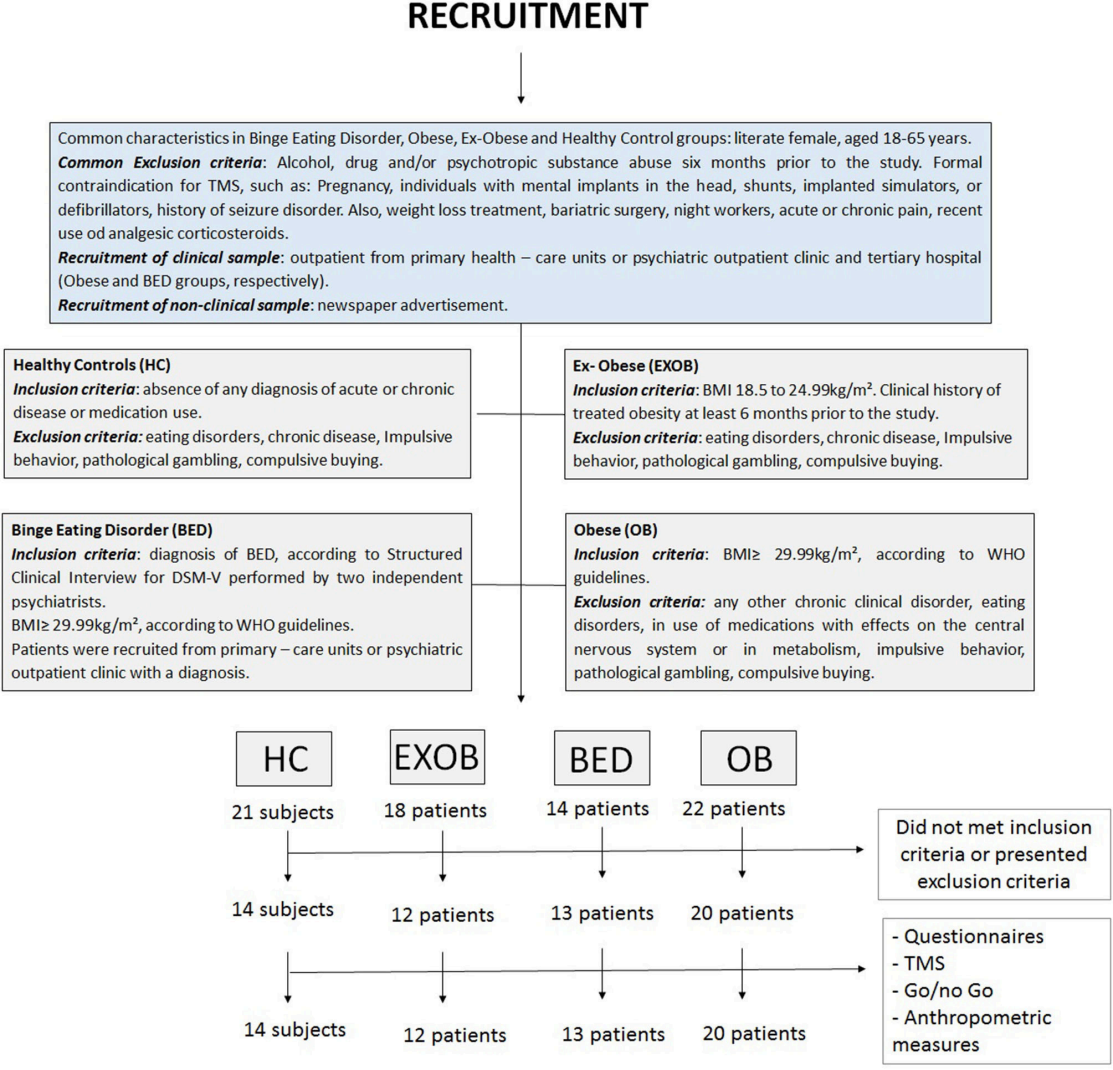
Flow of the study.

In addition, to assure that HC did not present any kind of impulsivity behavior, we applied the settled cutoffs to the Barratt Impulsiveness Scale (BIS-11) (16), the South Oaks Gambling Screen (SOGS) (17), and The Richmond Compulsive Buying Scale (RCBS) (18) as exclusion criteria. The inclusion and exclusion criteria pertaining to each one of the four groups (obese, BED, ex-obese, and HC) are detailed in Figure 1.

### Instruments and Assessment

#### Outcomes

The primary outcome was the motor cortex inhibitory function indexed by CSP. Secondary outcomes were SICI, ICF, response inhibition through the Go/No-go paradigm, and eating psychopathology by standardized questionnaires [Three-Factor Eating Questionnaire (TFEQ), Eating Disorder Examination Questionnaire (EDE-Q), and Binge Eating Scale (BES)].

#### Dependent Variables

##### Cortical Excitability Measures

*(a) TMS measures:* The researchers used a MagProX100 stimulator with a figure-eight coil (MagVenture Company, Lucernemarken, Denmark) to access cortical excitability on the left primary motor cortex (M1). Subjects were placed in a comfortable chair and informed about all the procedures, including possible sensations that might be experienced during TMS measures. Surface electromyography (EMG) electrodes were placed on the right first dorsal interosseous (FDI) muscle and its tendon. Each participant had their hot spot identified on left M1 positioning the coil at a 45° angle to the sagittal line tangential to the scalp, marking the individual site with a soft-tipped pen. The same researcher performed all TMS assessments to reduce variability. The following parameters were used to settle the TMS measures:

-*Motor threshold (MT)*: MT was obtained at rest (rMT), and it is a measure that reflects the excitability of the membrane potential of pyramidal neurons in M1 (19), and it is defined as the lowest stimulus needed to induce 50% of the FDI muscle-evoked potentials (MEPs). To settle MT measure, the minimum amplitude of 50 μV in 5 of 10 successive trials was performed, followed by single-pulse TMS with an intensity of 130% of MT to record 10 MEPs (20).

-*CSP:* CSP has been associated with an inhibitory network influenced by GABA-B receptors (21). CSP was recorded in milliseconds (ms) using an intensity of 130% of MT during FDI muscle activity measured on a dynamometer set to ~20% of the maximal force (22). The average of 10 sequential measures of MEP/CSP-rectified traces was then recorded, which makes it possible to visualize the voluntary EMG activity level at baseline (i.e., prior to the TMS pulse). Thus, the cessation of the CSP can be demarcated more precisely by the return of voluntary EMG activity relative to the tonic baseline EMG level, as the second method described by Rossini (20, 23).

*-SIC and ICF*: SICI mainly reflects GABA-A receptor-mediated inhibitory function (24, 25), while ICF denotes excitability of excitatory neuronal circuits in motor cortex, which are at least in part dissociable from the SICI network (26) and mostly mediated through the glutamatergic N-methyl-D-aspartate receptor (NMDA) (27), according to pharmacological experiments (26). It is conceivable that ICF is a net facilitation mainly comprising facilitation and weaker inhibition (28).

The TMS protocol to measure SICI and ICF used a total of 30 randomized paired-pulse trials, 10 for each measure (SICI, ICF, and control stimuli) with an interstimulus interval (ISI) to evaluate the SICI equal to 2 and 12 ms for ICF, respectively. The first individual conditioning stimulus was set at 80% of the MT, while the test stimulus was set at 130% (29, 30).

**Figure 2:**
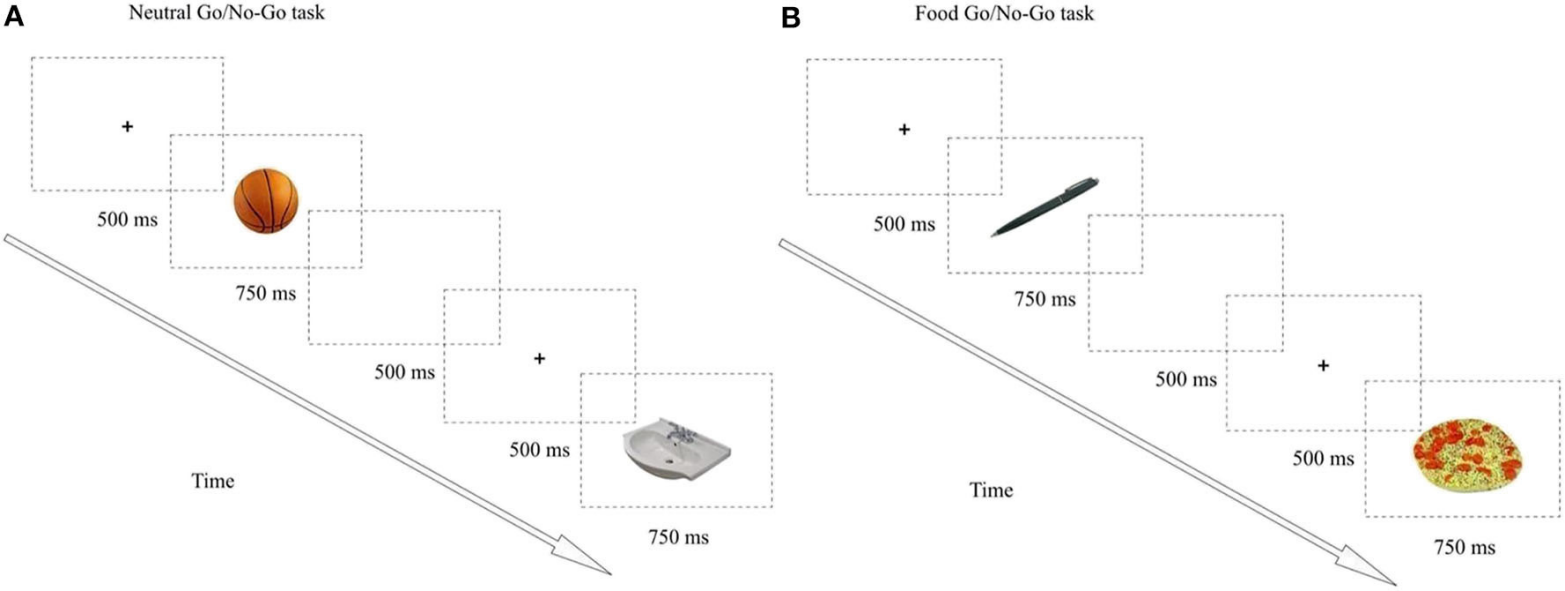
The Go/No-go paradigm. **(A)** Neutral task, **(B)** Food task. Each task had 100 trials divided in two blocks of 50. A total of 200 trials were presented (Neutral + Food). Images were presented using a ratio of 80% “go” to 20% “no-go” trials to create a prepotent “go” response. Each trial was presented for 750 ms and was separated by a blank screen for 500 ms and preceded by a fixation point for 500 ms. The go and no-go categories were presented in a pseudorandomized order, with 3, 4 or 5 go trials in between every no-go trials. Presentation order of the food and neutral tasks was fully counterbalanced.

*(b) Go/No-go paradigm:* It was performed to assess the response inhibition, the secondary outcome component of cognitive control in this study. The neutral and food-based Go/No-go tasks were designed and run using E-Prime™ software (Psychology Software Tools, Inc., Sharpsburg, PA, USA) by a psychologist. The images selected to compose the stimuli in this experiment were gathered from the Bank of Standardized Stimuli (BOSS), a free standardized set of visual stimuli, consisting of a normative database organized by category, familiarity, visual complexity, object agreement, and viewpoint agreement, and therefore, equivalent in both valence and perceptual characteristics (31). In the neutral-based task, the target (Go) stimulus was the toiletries images, while the non-target (No-go) stimulus was the images of sports equipment. In the food-based task, the target (Go) stimulus was images of neutral office supplies, while the non-target (No-go) stimulus was the images of high-fat and/or high-sugar foods. Distinct target image (Go) stimulus was selected to neutral and food tasks, to reduce the likelihood of an inductive bias due to a learning algorithm related to repetitive practice effects (Figure 2).

All stimuli were displayed in the center of a computer screen for 500 ms/each. The ISI is 1,000 ms (trial length = 1,500 ms). A fixation cross was displayed in the center of the screen during the ISI. Subjects were instructed to continuously verify a series of stimuli presented individually centered on the computer screen. They had to answer as fast as possible by pressing the keyboard space bar whenever a target (Go) stimulus was presented (80% of trials) and not answer to an infrequently presented not target [No-go stimulus (20% of trials)]. The presentation order was fully counterbalanced. The measure of interest was the number of commission errors (the number of incorrect answers performed during the No-go trials) (11). The entire task consisted of 200 trials divided into two 100 trial runs and the last 750 ms each.

##### Assessment of Eating Psychopathology

(c) Eating behavior psychopathology:

*-TFEQ:* It covers three eating-related concepts: dietary restraint, disinhibition, and susceptibility to hunger (32). It is a well-rated measure in which the dietary restraint refers to the tendency to consciously restrict food intake as a means of controlling weight, the disinhibition refers to a tendency to overeat in response to negative emotional states or the presence of highly palatable foods, and the hunger subscale assesses susceptibility to feelings of hunger.

*-EDE-Q:* It is a 28-item composed of four subscales and a global score that reflects the severity of eating disorder psychopathology (33).

*-BES*: It consists of a 16-item Likert scale, in which eight of them describe the behavioral manifestations and the others describe the feelings and cognition related to the binge eating episodes. A translated, adapted, and certified version in Brazilian Portuguese was applied to assess the severity of the binge eating episodes (34). The scores range from 0 to 46. A cutoff of equal to or >18 indicates the presence of binge eating.

*(d) Measures of hunger, satiety, and appetite:* According to a previous protocol designed by Jauch-Chara et al. (35), we provided a 10-point numerical rating scale [from 0 (any desire or need regard eating) to 10 (very prominent desire or need regard eating)] in which the subjects should score their hunger, feeling of hunger, satiety, and appetite to specific (sweet and savory food) and unspecific food in the last 24 h before the study assessment (35).

*(e) Standardized questionnaire:* it was used to assess demographic and medical data. Patients were requested to provide information about their age, sex, level of education, marital status, and lifestyle habits. They also provided information about their health status, including clinical and psychiatric diagnosis.

### Independent Variables

#### Anthropometry, Psychological, Demographic, and Clinical Characteristics

##### Anthropometric measures

Height was measured in centimeters (cm) using a height scale (Sanny, 14024) with the subject standing bare feet and with a normal straight posture. Weight was measured in kilograms (kg) using a weight scale (Toledo, 2096 PP). Obese and lean individuals were diagnosed according to the WHO criteria (BMI > 29.99 kg/m^2^ and between 18.5 and 24.99 kg/m^2^, respectively).

##### Psychiatric diagnosis

It was based on the Structured Clinical Interview for DSM-V (SCID) applied by two independent trained psychiatrists. This instrument consists of a semi-structured diagnostic interview created from DSM-V. The answers identify the presence or absence of the symptoms, scored according to the judgment of the reviewer. It is composed of 10 modules, which can be used in a combined or independent way (2012). In the study, the “A” module was used to diagnose mood episodes (major depressive disorders). The translation and adaptation of this clinical interview into the Portuguese language present, in general, good reliability for mood disorders (36).

##### Psychological state and sleep quality

All instruments used were validated for the Brazilian population and the assessments were conducted by two trained reviewers. The following tools were applied: Beck Depression Inventory-II (BDI-II) (37), Pittsburgh Sleep Quality Index (PSQI) (38), and State-Trait Anxiety Inventory (STAI) (39).

##### Demographic and clinical characteristics

Subjects were asked about current and previous (6 months prior to the study) use of psychotropic drugs. Licit and illicit psychotropic drugs were assessed by a standardized questionnaire, which also includes medical comorbidities and demographic data. Participants in both HC and Ex-obese groups were carefully screened to be drug-naive. Besides, they were taught not to take any medication, caffeine, or any stimulant drinks at least 6 h prior to the TMS assessment. Subjects from the BED and Obese groups, due to ethical issues, were instructed to sustain their prescribed medicines and they were, as well, taught not to take any additional medications, caffeine, or any stimulant drinks at least 6 h prior to the TMS assessment.

#### Efforts to Address Potential Sources of Bias

In order to reduce assessment bias, a trained researcher applied the psychiatric diagnosis based on the SCID-V and the instruments about eating behavior and clinical and demographic characteristics. To reduce the variability, the same researcher performed all TMS and Go/No-go task assessments.

### Study Size

A sample size of 60 participants were estimated for type I and type II errors of 0.05 and 0.20, respectively, and anticipating partial η^2^ of 0.25 for multiple regression analysis, which allows for two predictors (the group of eating disorder and use of psychotropics medication). It was calculated using the *post-hoc* statistical power calculator for hierarchical multiple regression at https://www.danielsoper.com/statcalc/calculator.aspx?id=17.

### Statistical Analysis

Shapiro–Wilk's test was performed to test normality. Descriptive statistics were used to summarize the main characteristics of the sample. ANOVA was performed to compare the four groups in the univariate analysis regarding cortical excitability and eating disorder psychopathology. MANCOVA was used to test the differences between groups (BED, obese, ex-obese, and HC) on the multiple outcomes controlled for psychotropic drugs. The dependent variables included in the MANCOVA were cortical excitability (CSP, SICI, and ICF), inhibitory control, and eating behavior (secondary outcomes).

Commission errors on “No-go” trials in the neutral-based Go/No-go task served as the gold standard for behavioral inhibition in these analyses. Performance differences on the neutral vs. food Go/No-go tasks were analyzed through GLM.

To analyze the correlation between the CSP, eating behavior, and the Go/No-Go task, Pearson's correlation analysis was used. All analyses were adjusted by multiple comparisons using the Bonferroni's multiple comparison test. To analyze the data, we used the software SPSS version 22.0 (SPSS, Chicago, IL, USA). Statistical significance was set at the 0.05 level for all analyses.

## Results

### Socio-Demographic, Clinical, and Psychological Characteristics of the Sample

Demographic, clinical, and psychiatric characteristics are summarized in Table 1. Univariate analysis evidenced that the BED group presented poor quality sleep compared to the HC and ex-obese groups. Subjects with BED showed higher levels of depressive symptoms compared to the other three groups.

**Table 1:** Demographic and clinical characteristics.

	**Obese (*n* = 20)**	**Binge eating disorder (*n* = 13)**	**Ex-obese (*n* = 12)**	**Health controls (*n* = 18)**	***P***
**Demographic**					
Age (years)	31 (±7.25)	26.42 (±4.27)	30.91 (±7.66)	32.17 (±7.85)	0.173
BMI^ȼ^ (kg/m^2^)	33.05 (±3.99)^b^	33.15 (±3.74)^b^	23.41 (±1.62)^a^	21.33 (±1.98)^a^	<0.001
Obesity I	14	8	–	–	–
Obesity II	3	3	–	–	–
Obesity III	3	1	–	–	–
Years of education	14.68 (±3.42)	15.04 (±3.24)	16.88 (±5.63)	16.53 (±2.56)	0.295
Employed (yes/no)	20/0	13/0	12/0	14/1	–
Alcohol use (yes/no)	13/7	9/4	9/3	14/1	–
**Clinical and psychiatric**					
Use of psychotropic medication					
Selective serotonin reuptake inhibition SSRTIs (yes/no)	3/17	7/6	0/0	0/0	–
Other antidepressants (yes/no)	1/19	5/10	0/0	0/0	–
Psychiatric disorder according to the SCID-V					
Major depressive episode	2/18	5/13	0/0	0/0	–
Bipolar disorder	1/19	1/13	0/0	0/0	–
Generalized anxiety disorder	5/15	8/13	2/12	0/0	–
Panic disorder	0/0	2/13	0/0	0/0	–
Attention deficit hyperactivity disorder	1/19	1/13	0/0	0/0	–
Beck Depression Inventory—BDI-II	10.15 (±8.45)^a^	25.15 (±11.02)^b^	11.08 (±9.85)^a^	8.44 (±7.60)^a^	<0.001
BDI-II (score > 10)	8	12	5	–	
State-Trait Anxiety Inventory					
STAI—State	22.75 (±6.48)	28.38 (±8.95)	25.0 (±12.84)	29.6 (±9.73)	0.146
STAI—Trait	21.05 (±4.47)	25.0 (±3.93)	23.41 (±4.92)	22.46 (±5.18)	0.123
Pittsburgh Sleep Quality Index (PSQI)	11.05 (±7.44)^a, b^	17.07 (±8.56)^b^	8.83 (±4.52)^a^	8.20 (±4.49)^a^	0.004
Poor Sleep—PSQI (score > 5)	15	12	10	9	
Binge Eating Scale	10.50 (±5.23)^a^	23.38 (±5.70)^b^	10.91(±7.94)^a^	7.07(±5.40)^a^	<0.001
International Physical Activity Questionnaire (MET)	4,407.15 (±4,768.80)	1,801.92 (±2,321.67)	6,604.66 (±8,337.37)	4,678.85 (±4,955.10)	0.177

*Data are presented as average and standard deviation (n = 59)*.

ȼ *Body mass index. Comparisons using ANOVA. Post-hoc differences among groups are indicated via superscript letters*.

### Univariate Analysis

#### Assessment of Cortical Excitability According to Groups

The cortical excitability parameters measured by TMS according to eating disorder group are presented in Table 2. It was observed that the BED group compared to obese, ex-obese, and HC displayed larger CSP (*F* = 2.359; *P* = 0.031). There were no significant differences among groups in the other cortical excitability measures (SICI and ICF).

**Table 2:** Cortical excitability measures assessed by the TMS.

	**Obese (*n* = 20)**	**Binge eating disorder (*n* = 13)**	**Ex-obese (*n* = 12)**	**Healthy control (*n* = 14)**	***F***	***P***
Motor threshold—MT (%)	44.5 (±6.9)	47.0 (±5.3)	47.8 (±5.0)	45.5 (±7.7)	0.571	0.637
Motor evocate potential—MEP (mV)	335.85 (±371.78)	693.62 (±1,083.13)	853.75 (±593.62)	584.55 (±710.90)	1.444	0.202
Intracortical Facilitation—ICF (mV)	1.45 (±2.29)	1.72 (±1.95)	2.28 (±1.88)	1.65 (±1.34)	0.489	0.858
Short Intracortical Inhibition—SICI (mV)	1.04 (±2.18)	0.64 (±0.69)	0.45 (±0.42)	0.22 (±0.22)	0.662	0.722
Cortical Silent Period—CSP (ms)	95.21 (±15.76)^a^	118.16 (±17.75)^b^	94.22 (±20.82)^a^	93.79 (±27.30)^a^	2.350	0.031

*Data are presented as average and standard deviation (n = 59). Different superscripts (a, b) indicate significance. To compare means, the analysis of variance (ANOVA) was used. Difference among groups after post hoc analysis adjusted by Bonferroni (P < 0.05)*.

### Multivariate Analysis—Primary Outcome

#### Cortical Excitability Measures According to Eating Disorder Diagnosis

A MANCOVA model that was constructed with the cortical excitability parameters (SICI, ICF, and CSP) as dependent variables, eating disorders (factor) as independent variables, and the use of psychotropic medications as a covariate is presented in Table 3. The analysis revealed a significant difference between the groups (Hotelling's Trace = 0.64, *F* = 2.436, *P* = 0.007). The BED group presented higher CSP than the three other groups. The use of psychotropic medications did not correlate with the SICI, ICF, and CSP.

**Table 3:** Primary outcome—multivariate linear regression model of the cortical excitability measures indexed by the CSP, SICI, and ICR according to groups of eating disorder (*n* = 59).

**(A) Main effects**						
**Dependent Variables**	**Type III Sum of Squares**	**df**	**Mean Square**	***F***	***P***	**Partial Eta Squared**
**Corrected model**						
Short intracortical inhibition [(SICI, ratio: SICI/test stimulus)]	10.017^a^	8	1.25	0.66	0.722	0.10
Cortical Silent period [(CSP, ratio: CSP/test stimulus)]	7,825.86^b^	8	978.23	2.35	0.031	0.27
Intracortical facilitation [(ICF), ratio: ICF/test stimulus]	15.44^c^	8	1.93	0.48	0.858	0.07
**(B) Beta coefficients**						
	**B**	**SEM**	***t***	***P***		**95% CI**
**Primary outcome**						
**Dependent variable: Cortical Silent Period [(CSP), ratio: CSP/test stimulus]**						
Intercept	111.381	28.590	3.896	<0.001		(53.95 to 168.80)
Obese	17.533	11.948	1.467	0.149		(−6.47 to 41.53)
Healthy controls	−3.306	8.205	−0.403	0.689		(−19.79 to 13.17)
Ex-obese	0^reference^					
Binge Eating Disorder	42.480	13.151	3.230	0.002		(16.06 to 68.90)
Use of psychotropic medication	−8.805	10.412	−0.846	0.402		(−29.71 to 12.10)
**Secondary outcome**						
**Dependent variable: Short intracortical inhibition [(SICI), ratio: SICI/test stimulus]**						
Intercept	−0.360	1.927	−0.187	0.853		(−4.23 to 3.51)
Obese	0.381	0.805	0.474	0.638		(−1.24 to 2.00)
Healthy controls	−0.217	0.553	−0.393	0.696		(−1.33 to 0.89)
Ex obese	0^reference^					
Binge Eating Disorder	−0.001	0.886	−0.002	0.999		(−1.78 to 1.78)
Use of psychotropic medication	−0.810	0.702	−1.155	0.254		(−2.22 to 0.60)
**Dependent variable: Intracortical facilitation [(ICF), ratio: ICF/ test stimulus]**						
Intercept	−0.169	2.783	−0.061	0.952		(−5.76 to 5.42)
Obese	−2.169	1.163	−1.865	0.068		(−4.50 to 0.17)
Healthy controls	−0.409	0.799	−0.512	0.611		(−2.01 to 1.19)
Ex obese	0^reference^					
Binge Eating Disorder	−2.010	1.280	−1.570	0.123		(−4.58 to 0.56)
Use of psychotropic medication	−0.701	1.014	−0.692	0.492		(−2.74 to 1.33)

a *R^2^ = 0.96 (Adjusted R^2^ = −0.049)*.

b *R^2^ = 0.273 (Adjusted R^2^ = 0.157)*.

c *R^2^ = 0.073 (Adjusted R^2^ = −0.076)*.

### Multivariate Analysis—Secondary Outcomes

#### Assessment of the Relationship Between CSP and Response Inhibition Through the Go/No-Go Paradigm With Eating Behavior Psychopathology According to the Groups

A MANCOVA model, with the cortical silent period (primary outcome), Go/No-Go paradigm (commission errors from neutral and food task), and Eating Behavior (EDE-Q global score and BES) as dependent variables, was used. The BED group presented higher CSP than the other three groups. Also, the BED group presented higher scores in the BES than the other three groups, which was positively influenced by the BDI-II (score). The HC group presented a lower global score in EDE-Q than the other groups, and this finding was negatively correlated with depressive symptoms assessed through BDI-II. However, we did not find differences between groups in the Go/No-Go task.

### Exploratory Analysis

#### Correlation Among CSP, Eating Behavior, Depressive Symptoms, and Sleep Quality

**Figure 3:**
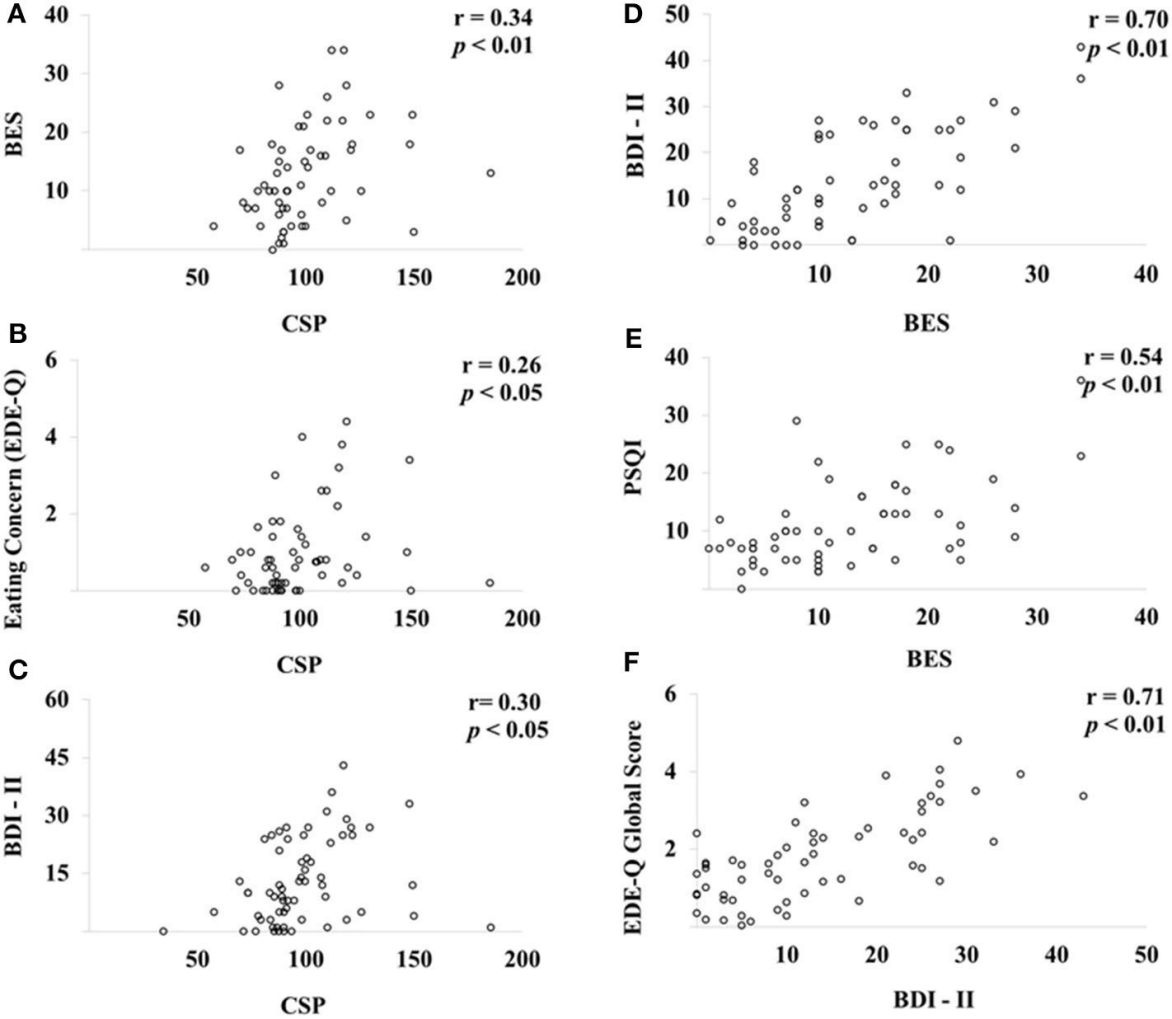
Pearson's correlation analysis among CSP, eating behavior, and inhibitory control through the Go/No-Go paradigm. Scatter plots of CSP, eating behavior, and Go/No-Go paradigm (*n* = 60). BES, Binge Eating Scale; CSP, cortical silent period; BDI-II, Beck Depression Inventory; PSQI, Pittsburgh Sleep Quality Index; EDE-Q, Eating Disorder Examination Questionnaire. The CSP was positively correlated with BES **(A)**, Eating Concern **(B)** and BDI-II **(C)**, while the BES was positively correlated to BDI-II **(D)** and PSQI **(E)**. BDI-II was positively associated with the EDE-Q global score **(F)**.

Pearson's correlation analysis coefficients among CSP, eating behavior, and inhibitory control through the Go/No-Go paradigm area are presented in Table 4. The CSP was positively correlated with BES (*r* = 0.34, *P* < 0.01), Eating Concern (*r* = 0.26, *P* < 0.05), and BDI-II (*r* = 0.30, *P* < 0.05), while the BES was positively correlated to BDI-II (*r* = 0.70, *P* < 0.01) and PSQI (*r* = 0.54, *P* < 0.01). BDI-II was positively associated with the EDE-Q global score (*r* = 0.71, *P* < 0.01) (Figure 3).

**Table 4:** Multivariate linear regression model of the cortical inhibition and eating psychopathology according to clinical and non-clinical groups (*n* = 59).

**(A) Main effects**						
**Dependent variables**	**Type III Sum of Squares**	**df**	**Mean Square**	***F***	***P***	**Partial Eta Squared**
**Corrected model**						
Cortical Silent Period	5,803.158^e^	5	1,160.632	2.727	0.029	0.208
Binge Eating Scale	2,628.373^a^	5	525.675	20.105	<0.001	0.659
Global Score—Eating Disorder Examination Questionnaire	44.151^b^	5	8.830	14.342	<0.001	0.580
Commission errors neutral	13.409^c^	5	2.682	0.965	0.448	0.085
Commission errors food	23.836^d^	5	4.767	1.950	0.102	0.158
**(B) Beta coefficients**						
	**B**	**SEM**	***t***	***P***		**95% CI**
**Primary outcome**						
**Dependent variable: Cortical Silent Period [(CSP), ratio: CSP/test stimulus]**						
Intercept	96.618	7.316	13.206	<0.001		81.93 to 111.29
Healthy controls	0.635	8.282	0.077	0.939		−15.98 to 11.25
Obese	2.082	7.626	0.273	0.786		−13.22 to 17.38
Binge Eating Disorder	25.702	9.491	2.708	0.009		6.65 to 44.75
Ex-obese	0^a^	.	.	.		.
Beck Depression Inventory	0.130	0.330	0.394	0.695		−0.53 to 0.79
Pittsburgh Sleep Quality Index	−0.435	0.463	−0.940	0.351		−1.34 to 0.49
**Secondary outcome**						
**Dependent variable: Binge Eating Scale**						
Intercept	6.298	1.813	3.473	0.001		2.66 to 9.94
Healthy controls	−3.332	2.053	−1.623	0.111		−7.45 to 0.79
Obese	−0.432	1.890	−0.229	0.820		−4.22 to 3.36
Binge Eating Disorder	7.011	2.352	2.980	0.004		2.29 to 11.73
Ex-obese	0^a^	.	.	.		.
Beck Depression Inventory	0.308	0.082	3.760	<0.001		0.14 to 0.37
Pittsburgh Sleep Quality Index	0.137	0.115	1.192	0.239		−0.09 to 0.37
**Dependent variable: Global score Eating Disorder Examination Questionnaire**						
Intercept	1.085	0.278	3.899	<0.001		0.52 to 1.64
Healthy controls	−0.756	0.315	−2.400	0.020		−1.38 to −0.12
Obese	−0.054	0.290	−0.187	0.852		−0.63 to 0.53
Binge Eating Disorder	0.107	0.361	0.297	0.768		−0.61 to 0.83
Ex-obese	0^a^	.	.	.		.
Beck Depression Inventory	0.067	0.013	5.307	<0.001		0.04 to 0.09
Pittsburgh Sleep Quality Index	0.000	0.018	0.024	0.981		−0.03 to 0.04
**Dependent variable: Commission errors neutral**						
Intercept	2.993	0.591	5.063	<0.001		1.80 to 4.17
Healthy controls	−1.018	0.669	−1.521	0.134		−2.36 to 0.33
Obese	0.149	0.616	0.243	0.809		−1.08 to 1.39
Binge Eating Disorder	0.411	0.767	0.536	0.594		−1.128 to 1.95
Ex-obese	0^a^	.	.	.		.
Beck Depression Inventory	−0.003	0.027	−0.115	0.909		−0.06 to 0.05
Pittsburgh Sleep Quality Index	−0.024	0.037	−0.631	0.531		−0.09 to 0.05
**Dependent variable: Commission errors food**						
Intercept	1.958	0.554	3.530	0.001		0.84 to 3.07
Healthy controls	−0.866	0.628	−1.380	0.174		−2.12 to 0.39
Obese	−0.182	0.578	−0.314	0.755		−1.34 to 0.98
Binge Eating Disorder	0.032	0.719	0.044	0.965		−1.41 to 1.48
Ex-obese	0^a^	.	.	.		.
Beck Depression Inventory	−0.044	0.025	−1.746	0.087		−0.09 to.007
Pittsburgh Sleep Quality Index	0.079	0.035	2.239	0.029		0.008 to 0.15

a *R^2^ = 0.659 (Adjusted R^2^ = 0.626)*.

b *R^2^ =0.580 (Adjusted R^2^ = 0.539)*.

c *R^2^ =0.085 (Adjusted R^2^ = − 0.003)*.

d *R^2^ =0.158 (Adjusted R^2^ = 0.077)*.

e *R^2^ =0.208 (Adjusted R^2^ = 0.132)*.

## Discussion

The innovation of these findings is to indicate that BED may be associated with a dysfunction in the GABAergic system as indicated by a longer CSP in this group compared to the other three. This finding is relevant in a physiological field, and it may be clinically relevant to investigate new therapeutic strategies in BED, mainly because it is one of the latest eating disorders formally recognized in the DSM-5 (1). This finding is supported by earlier studies that revealed that the CSP is an index of intracortical inhibition, as shown in a previous study that assessed the GABAB inhibition processes (21). We hypothesized that the mechanisms underlying the CSP could participate in the generation of surround inhibition and disengage pathways involved in the eating control. Although underpinning mechanisms of our results related to longer CSP in BED are not clear, according to earlier studies, the CSP reflects the inhibitory activity in the M1 (40–42), which is modulated by several cortical and subcortical systems (43). Our hypothesis that BED is associated with an amplified GABAB-mediated inhibitory activity in the primary motor cortex (M1) is supported by pharmacological studies with IV selective GABAB agonist baclofen, which revealed the augmentation of CSP length followed its infusion (44).

It is thought that GABABR signaling in the mesolimbic system regulates hedonic food consumption, whereas the hypothalamic neurons play an essential role in the homeostatic regulation of energy balance. Based on this rationale, experimental (45, 46) and clinical (47, 48) data have confirmed the effectiveness of baclofen in the reduction of binge eating. Regarding mechanism, baclofen acts on presynaptic GABAB receptors by modulating G-protein-gated inwardly rectifying potassium (GIRK/Kir3) channels to inhibit cortico-mesolimbic dopamine neurotransmission (49).

Therefore, we hypothesize that the augmented GABAB activity, indexed by prolonged CSP, found in BED individuals could reflect the enhancement of certain inhibitory phenomena in patients with this disorder. Indeed, they presented a modified balance between excitability and inhibition of neural networks, which chronically shifts toward the overt hyperexcitability. This assumption can be consistent with the loss of control overeating. Following in this line, our findings are supported by cumulative evidence in epilepsy research that also verified a robust amplification of the CSP in untreated patients with generalized and partial epilepsies when compared to HC (50, 51). Hence, our results, as well as previous epilepsy research, point out that the large CSP might designate a hyperactivation of inhibitory neural circuits. Besides, it is conceivable that the higher CSP length in BED individuals displays hyperexcitability of inhibitory circuits as a consequence of unsuitable neuroplastic changes in the attempt to restore homeostasis.

Interestingly, the role of GABABR in corticostriatal neurons has been explored in a knockout (KO) model, which revealed the increase in binge-like feeding of high-fat diet (HFD), while the administration of baclofen was able to suppress it presumably via the mesolimbic pathway. In addition, they have shown that GABABR signaling in the mesolimbic system does not affect energy balance, concluding that the mesolimbic system regulates binge-like eating of HFD and providing a mechanism by which the GABABR signaling suppresses palatable food consumption (52). However, it is essential to emphasize that GABABRs were also knocked out in mPFC and OFC, which are known critical areas in decision-making (53) and, therefore, could also be implicated in this binge eating model. Notwithstanding, it remains unclear which neurotransmitter is critical for GABABR signaling to suppress binge eating behavior.

In line with the abnormal cortical excitability in the BED group, CSP was correlated with scores of BES and EDE-Q. Eating behavior assessed by the BES and EDE-Q was correlated with the BDI-II. Psychiatric comorbidity rates are significantly higher among obese individuals with BED compared to those without BED (1), and psychiatric comorbidity is linked to the severity of binge eating (1). Indeed, comorbid depressive symptoms in individuals with BED have been correlated with greater traits of impulsivity and sensitivity to punishment (54). Taken together, the high comorbidity between BED and MDD might be explained through abnormalities found in the dopaminergic and serotonergic function, which strengthens the comorbidity differences compared to those obese individuals without BED diagnosis (55–57). Besides, eating disorders such as BED are associated with more reduced sleep quality and, notably, worsening binge eating (58).

Contrary to our hypothesis, we have not found inhibitory control deficits in response to high-calorie foods in the Go/No-go task among the groups. Obesity and BED partake a disruption in the executive function related to impulsivity, which is not entirely supported by previous research (59). A neuroimaging study found reduced activation in the prefrontal cortex (inferior frontal gyrus, ventromedial prefrontal cortex, and dorsolateral prefrontal cortex) during an inhibitory control task in obese participants compared to the control task on lean participants and there were no differences in inhibitory control performance task (5, 60), suggesting that obese individuals might have difficulty in maintaining inhibitory control, rather than a general impairment, and the maintenance also depends on external factors like motivation state (61). It is important to emphasize that the performance in tasks assessing inhibitory control, especially those addressed by food-associated tasks, is inconsistent since negative results in inhibitory control deficits have been previously reported (62). Remarkably, Loeber et al. have underpinned factors related to these conflicting results. The authors have provided evidence that group differences in inhibitory control were moderated by the interaction of restrained eating and mood (63). In addition, the higher commission errors related to food stimuli performed by the BED group occurred when individuals were restrained eaters and in a very positive mood at the time of testing, which, somehow, might explain why our study did not observe deficits in the Go/No-go task among groups (63).

Our findings could contribute to expanding the comprehension of the pathophysiological substrates of BED, suggesting dysfunction of GABAergic inhibitory interneurons in prefrontal cortices since cumulative TMS studies provided evidence that CSP is influenced by the inhibitory function of cortical or subcortical structures (64). Despite the contribution of our results in the comprehension of changes in measures related to neuroplasticity in the two disorders, the correlational nature does not allow a causality relationship.

This study presents some limitations: First, TMS consists of an indirect neurophysiological measure intended to assess the activity of a neurotransmitter system. Though we have properly chosen ISI, it is important to point out that even if SICI at 2 ms and ICF at 12 ms resulted in normal BED, it does not allow stating its normality at other ISIs, since the paired-pulse paradigm typically provides a stimulus–response curve of intracortical excitability at different ISIs for both inhibition (e.g., 2, 3, and 5 ms) and facilitation (e.g., 10, 12, and 15 ms). In line with that, it must be considered that we have chosen a protocol to elicit CSP during a muscle activity settled to approximately 20% of the maximal force, and not to ~50% as recommended, and that can explain the variability of responses between studies. However, we believe that setting 20% of the maximal force could provide a more accurate measure of GABA-mediated excitability of inhibitory neural circuits, as recently described (65). Second, psychiatric disorders remain a potential confounding factor, especially because both severity of depressive symptomatology and poor sleep quality, which are frequently comorbid conditions found in BED patients, may have affected the findings, since both of them can alter cortical excitability, including CSP length (66, 67). On the other hand, it is important to point out that we have controlled the analysis for these potentially confounding variables, including anxiety levels, depressive symptoms, eating psychopathology, and psychiatric diagnosis. Third, we must address the effect of psychotropic medicines under cortical excitability because the regular prescription of these medicines deliberates the proper treatment of BED. Nevertheless, it is critical to mention that different changes in cortical excitability produced using psychotropic medications might produce distinctive outcomes in acute and long-term use. Fourth, GABAB neurophysiological deficits may be closely related to the pathophysiology of major depressive disorder (MDD), which is a frequent comorbid disorder in individuals with BED, and in a clinical study, we cannot dissociate these conditions, which are comorbidly and closely related with BED. Therefore, the difference among groups in depressive symptoms could constitute a confounding factor in the relationship between CSP and BED. However, it is essential to emphasize that we carefully included in the analysis the potential confounders related to each dependent variable analyzed, such as psychiatric diagnosis and depressive symptoms.

## Data Availability Statement

The original contributions presented in the study are included in the article, further inquiries can be directed to the corresponding author/s.

## Ethics Statement

This study was approved by the Ethics Committee Board of the Hospital de Clínicas de Porto Alegre (Institutional Review Board IRB 0000921) and the participants signed the consent form before participating in this study.

## Author Contributions

All authors made a significant contribution to (a) the study concept and design, data collection or analysis and interpretation of data, (b) drafting/revising the manuscript for important intellectual content, and (c) approval of the final version to be published.

## Conflict of Interest

The authors declare that the research was conducted in the absence of any commercial or financial relationships that could be construed as a potential conflict of interest.

## References

[1] American Psychiatry Association Diagnostic and Statistical Manual of Mental Disorder. Washington, DC; Arlington, VA, 5th ed (2013). 10.1017/69780890425596

[2] Smith DG Robbins TW. The neurobiological underpinnings of obesity and binge eating: a rationale for adopting the food addiction model. Biol Psychiatry. (2013) 73:804–10. 10.1016/j.biopsych.2012.08.026 23098895

[3] Volkow ND Wang GJ Tomasi D Baler RD. The addictive dimensionality of obesity. Biol Psychiatry. (2013) 73:811–8. 10.1016/j.biopsych.2012.12.020 23374642 PMC4827347

[4] Giel KE Teufel M Junne F Zipfel S Schag K. Food-related impulsivity in obesity and binge eating disorder—a systematic update of the evidence. Nutrients. (2017) 9:1170. 10.3390/nu9111170 29077027 PMC5707642

[5] Balodis IM Molina ND Kober H Worhunsky PD White MA Sinha R. Divergent neural substrates of inhibitory control in binge eating disorder relative to other manifestations of obesity. Obesity. (2013) 21:367–77. 10.1002/oby.20068 23404820 PMC3610836

[6] Stramba-Badiale C Mancuso V Cavedoni S Pedroli E Cipresso P Riva G. Transcranial magnetic stimulation meets virtual reality: the potential of integrating brain stimulation with a simulative technology for food addiction. Front Neurosci. (2020) 14:720. 10.3389/fnins.2020.00720 32760243 PMC7372037

[7] Cristina De Macedo I Soares De Freitas J Lucena I Torres S. The influence of palatable diets in reward system activation: a mini review. (2016) 2016:7238679. 10.1155/2016/7238679 27087806 PMC4818794

[8] Lavagnino L Arnone D Cao B Soares JC Selvaraj S. Inhibitory control in obesity and binge eating disorder: a systematic review and meta-analysis of neurocognitive and neuroimaging studies. Neurosci Biobehav Rev. (2016) 68:714–26. 10.1016/j.neubiorev.2016.06.041 27381956

[9] Córdova ME Schiavon CC Busnello FM Reppold CT. Nutritional and neuropsychological profile of the executive functions on binge eating disorder in obese adults. Nutr Hosp. (2017) 34:1448–54. 10.20960/nh.1151 29280663

[10] Lyu Z Zheng P Chen H Jackson T. Approach and inhibition responses to external food cues among average-weight women who binge eat and weight-matched controls. Appetite. (2017) 108:367–74. 10.1016/j.appet.2016.10.025 27789376

[11] Price M Lee M Higgs S. Food-specific response inhibition, dietary restraint and snack intake in lean and overweight/obese adults: a moderated-mediation model. Int J Obes. (2016) 40:877–82. 10.1038/ijo.2015.235 26592733 PMC4856731

[12] Brewer JA Potenza MN. The neurobiology and genetics of impulse control disorders: relationships to drug addictions. Biochem Pharmacol. (2008) 75:63–75. 10.1016/j.bcp.2007.06.043 17719013 PMC2222549

[13] Sinha R. Role of addiction and stress neurobiology on food intake and obesity. Biol Psychol. (2018) 131:5–13. 10.1016/j.biopsycho.2017.05.001 28479142 PMC6784832

[14] Klomjai W Katz R Lackmy-Vallée A. Basic principles of transcranial magnetic stimulation (TMS) and repetitive TMS (rTMS). Ann Phys Rehabil Med. (2015) 58:208–13. 10.1016/j.rehab.2015.05.005 26319963

[15] Jáuregui-Lobera I Martínez-Quiñones J V. Neuromodulation in eating disorders and obesity: a promising way of treatment? Neuropsychiatr Dis Treat. (2018) 14:2817–35. 10.2147/NDT.S180231 30464467 PMC6208872

[16] Malloy-Diniz LF Mattos P Leite WB Abreu N Coutinho G De Paula JJ Translation and cultural adaptation of Barratt Impulsiveness Scale (BIS-11) for administration in Brazilian adults. J Bras Psiquiatr. (2010) 59:99–105. 10.1590/S0047-20852010000200004

[17] Oliveira MPM Silva MTA Silveira DX da Validity Study of the South Oaks Gambling Screen (SOGS) among distinct groups of Brazilian gamblers. Rev Bras Psiquiatr. (2002) 24:170–6. 10.1590/s1516-44462002000400005

[18] Leite P Rangé B Kukar-Kiney M Ridgway N Monroe K Ribas Junior R. Cross-cultural adaptation, validation and reliability of the Brazilian version of the Richmond Compulsive Buying Scale. Rev Bras Psiquiatr. (2013) 35:38–43. 10.1016/j.rbp.2012.10.004 23567598

[19] Nielsen JF Norgaard P. Increased post-exercise facilitation of motor evoked potentials in multiple sclerosis. Clin Neurophysiol. (2002) 113:1295–300. 10.1016/S1388-2457(02)00153-0 12140010

[20] Rossini PM Burke D Chen R Cohen LG Daskalakis Z Di Iorio R. Non-invasive electrical and magnetic stimulation of the brain, spinal cord, roots and peripheral nerves: Basic principles and procedures for routine clinical and research application: an updated report from an I.F.C.N. Committee. Clin Neurophysiol. (2015) 126:1071–107. 10.1016/j.clinph.2015.02.001 25797650 PMC6350257

[21] Werhahn KJ Kunesch E Noachtar S Benecke R Classen J. Differential effects on motorcortical inhibition induced by blockade of GABA uptake in humans. J Physiol. (1999) 517:591–7. 10.1111/j.1469-7793.1999.0591t.x 10332104 PMC2269337

[22] Kobayashi M Pascual-Leone A. Transcranial magnetic stimulation in neurology. Lancet. (2003) 2:145–56. 10.1016/s1474-4422(03)00321-1 12849236

[23] Medeiros LF Caumo W Dussán-Sarria J Deitos A Brietzke A Laste G. Effect of deep intramuscular stimulation and transcranial magnetic stimulation on neurophysiological biomarkers in chronic myofascial pain syndrome. Pain Med. (2015) 17:122–35. 10.1111/pme.12919 26408420

[24] Ilić TV Meintzschel F Cleff U Ruge D Kessler KR Ziemann U. Short-interval paired-pulse inhibition and facilitation of human motor cortex: the dimension of stimulus intensity. J Physiol. (2002) 545:153–67. 10.1113/jphysiol.2002.030122 12433957 PMC2290644

[25] Cash RFH Noda Y Zomorrodi R Radhu N Farzan F Rajji TK. Characterization of glutamatergic and GABA_A_-mediated neurotransmission in motor and dorsolateral prefrontal cortex using paired-pulse TMS-EEG. Neuropsychopharmacology. (2017) 42:502–11. 10.1038/npp.2016.133 27461082 PMC5399228

[26] Ziemann U Lönnecker S Steinhoff BJ Paulus W. The effect of lorazepam on the motor cortical excitability in man. Exp Brain Res. (1996) 109:127–35. 10.1007/BF00228633 8740215

[27] Ziemann U Hallett M Cohen LG. Mechanisms of deafferentation-induced plasticity in human motor cortex. J Neurosci. (1998) 18:7000–7. 10.1523/jneurosci.18-17-07000.1998 9712668 PMC6792971

[28] Ziemann U. TMS and drugs. Clin Neurophysiol. (2004) 115:1717–29. 10.1016/j.clinph.2004.03.006 15261850

[29] Kujirai T Caramia MD Rothwell JC Day BL Thompson PD Ferbert A. Corticocortical inhibition in human motor cortex. J Physiol. (1993) 471:501–19. 10.1113/jphysiol.1993.sp019912 8120818 PMC1143973

[30] Pascual-Leone A Valls-Solé J Wassermann EM Hallett M. Responses to rapid-rate transcranial magnetic stimulation of the human motor cortex. Brain. (1994) 117:847–58. 10.1093/brain/117.4.847 7922470

[31] Brodeur MB Dionne-Dostie E Montreuil T Lepage M. The bank of standardized stimuli (BOSS), a new set of 480 normative photos of objects to be used as visual stimuli in cognitive research. PLoS ONE. (2010) 5:e10773. 10.1371/journal.pone.0010773 20532245 PMC2879426

[32] de Medeiros ACQ Yamamoto ME Pedrosa LFC Hutz CS. The Brazilian version of the three-factor eating questionnaire-R21: psychometric evaluation and scoring pattern. Eat Weight Disord. (2016) 22:169–75. 10.1007/s40519-016-0256-x 26860610

[33] Machado PPP Martins C Vaz AR Conceição E Bastos AP Gonçalves S. Eating Disorder Examination Questionnaire: psychometric properties and norms for the Portuguese population. Eur Eat Disord Rev. (2014) 22:448–53. 10.1002/erv.2318 25175299

[34] Freitas S Lopes CS Coutinho W Appolinario JC Translation and adaptation into Portuguese of the Binge-Eating Scale. Rev Bras Psiquiatr. (2001) 23:215–20. 10.1590/s1516-44462001000400008

[35] Jauch-Chara K Kistenmacher A Herzog N Schwarz M Schweiger U Oltmanns KM. Repetitive electric brain stimulation reduces food intake in humans. Am J Clin Nutr. (2014) 100:1003–9. 10.3945/ajcn.113.075481 25099550

[36] Del-Ben CM Vilela JAA de S Crippa JA Hallak JEC Labate CM Zuardi AW. Reliability of the structured clinical interview for DSM-IV—clinical version translated into Portuguese. Rev Bras Psiquiatr. (2001) 23:156–9. 10.1590/s1516-444620010003000089222432

[37] Gomes-Oliveira MH Gorenstein C Neto FL Andrade LH Wang YP. Validation of the Brazilian Portuguese Version of the Beck Depression Inventory-II in a community sample. Rev Bras Psiquiatr. (2012) 34:389–94. 10.1016/j.rbp.2012.03.005 23429809

[38] Bertolazi AN Fagondes SC Hoff LS Dartora EG da Silva Miozzo IC de Barba MEF. Validation of the Brazilian Portuguese version of the Pittsburgh Sleep Quality Index. Sleep Med. (2011) 12:70–5. 10.1016/j.sleep.2010.04.020 21145786

[39] Balle M Chachamovich E Paz M Hidalgo L Lucena I Caumo W. Evaluation of the structure of Brazilian State-Trait Anxiety Inventory using a Rasch psychometric approach. J Psychosom Res. (2010) 68:223–33. 10.1016/j.jpsychores.2009.09.013 20159207

[40] Fuhr P Agostino R Hallett M. Spinal motor neuron excitability during the silent period after cortical stimulation. Electroencephalogr Clin Neurophysiol Evoked Potentials. (1991) 81:257–62. 10.1016/0168-5597(91)90011-L 1714819

[41] Roick H von Giesen HJ Benecke R. On the origin of the postexcitatory inhibition seen after transcranial magnetic brain stimulation in awake human subjects. Exp Brain Res. (1993) 94:489–98. 10.1007/BF00230207 8359263

[42] Cincotta M Quartarone A Abbruzzese G Motor cortical and corticospinal measures in health and disease. In: Miniussi C., Paulus W., Rossini P. M., editors. Transcranial Brain Stimulation. CRC Press, (2012) 159–205. 10.1201/b14174-9

[43] Cincotta M Borgheresi A Guidi L Macucci M Cosottini M Lambruschini P. Remote effects of cortical dysgenesis on the primary motor cortex: evidence from the silent period following transcranial magnetic stimulation. Clin Neurophysiol. (2000) 111:1340–5. 10.1016/s1388-2457(00)00330-8 10904213

[44] Stetkarova I Kofler M. Differential effect of baclofen on cortical and spinal inhibitory circuits. Clin Neurophysiol. (2013) 124:339–45. 10.1016/j.clinph.2012.07.005 22877625

[45] Berner LA Bocarsly ME Hoebel BG Avena NM Baclofen suppresses binge eating of pure fat but not a sugar-rich or sweet-fat diet. Behav Pharmacol. (2009) 20:631–4. 10.1097/FBP.0b013e328331ba47 19752722 PMC3291953

[46] Czyzyk TA Sahr AE Statnick MA A model of binge-like eating behavior in mice that does not require food deprivation or stress. Obesity. (2010) 18:1710–7. 10.1038/oby.2010.46 20300082

[47] Broft AI Spanos A Corwin RL Mayer L Steinglass J Devlin MJ. Baclofen for binge eating: an open-label trial. Int J Eat Disord. (2007) 40:687–91. 10.1002/eat.20434 17647277

[48] Corwin RL Boan J Peters KF Ulbrecht JS. Baclofen reduces binge eating in a double-blind, placebo-controlled, crossover study. Behav Pharmacol. (2012) 23:616–25. 10.1097/FBP.0b013e328357bd62 22854310

[49] Cruz HG Ivanova T Lunn ML Stoffel M Slesinger PA Lüscher C. Bi-directional effects of GABAB receptor agonists on the mesolimbic dopamine system. Nat Neurosci. (2004) 7:153–9. 10.1038/nn1181 14745451

[50] Macdonell RA King MA Newton MR Curatolo JM Reutens DC Berkovic SF. Prolonged cortical silent period after transcranial magnetic stimulation in generalized epilepsy. Neurology. (2001) 57:706–8. 10.1212/wnl.57.4.706 11524485

[51] Cincotta M Giovannelli F Borgheresi A Tramacere L Viggiano MP Zaccara G. A meta-analysis of the cortical silent period in epilepsies. Brain Stimul. (2015) 8:693–701. 10.1016/j.brs.2015.04.008 25981158

[52] Tsunekawa T Banno R Yaginuma H Taki K Mizoguchi A Sugiyama M. GABAB Receptor signaling in the mesolimbic system suppresses binge-like consumption of a high-fat diet. iScience. (2019) 20:337–47. 10.1016/j.isci.2019.09.032 31610370 PMC6817655

[53] Hare TA O'Doherty J Camerer CF Schultz W Rangel A. Dissociating the role of the orbitofrontal cortex and the striatum in the computation of goal values and prediction errors. J Neurosci. (2008) 28:5623–30. 10.1523/JNEUROSCI.1309-08.2008 18509023 PMC6670807

[54] Carrard I Crépin C Ceschi G Golay A Van der Linden M. Relations between pure dietary and dietary-negative affect subtypes and impulsivity and reinforcement sensitivity in binge eating individuals. Eat Behav. (2012) 13:13–9. 10.1016/j.eatbeh.2011.10.004 22177390

[55] Majuri J Joutsa J Johansson J Voon V Alakurtti K Parkkola R. Dopamine and opioid neurotransmission in behavioral addictions: a comparative PET study in pathological gambling and binge eating. Neuropsychopharmacology. (2017) 42:1169–77. 10.1038/npp.2016.265 27882998 PMC5357051

[56] Majuri J Joutsa J Johansson J Voon V Parkkola R Alho H. Serotonin transporter density in binge eating disorder and pathological gambling: A PET study with [11C]MADAM. Eur Neuropsychopharmacol. (2017) 27:1281–8. 10.1016/j.euroneuro.2017.09.007 29032922

[57] Joutsa J Karlsson HK Majuri J Nuutila P Helin S Kaasinen V. Binge eating disorder and morbid obesity are associated with lowered mu-opioid receptor availability in the brain. Psychiatry Res Neuroimaging. (2018) 276:41–5. 10.1016/j.pscychresns.2018.03.006 29655552

[58] Mason TB Engwall A Mead MP Irish LA. Sleep and eating disorders among adults enrolled in a commercial weight loss program: associations with self-report and objective sleep measures. Eat Weight Disord. (2019) 24:307–12. 10.1007/s40519-019-00664-1 30852800

[59] Blume M Schmidt R Hilbert A. Executive functioning in obesity, food addiction, and binge-eating disorder. Nutrients. (2019) 11:54. 10.3390/nu11010054 30597858 PMC6356459

[60] Kessler RM Hutson PH Herman BK Potenza MN. The neurobiological basis of binge-eating disorder. Neurosci Biobehav Rev. (2016) 63:223–38. 10.1016/j.neubiorev.2016.01.013 26850211

[61] Lawyer SR Boomhower SR Rasmussen EB. Differential associations between obesity and behavioral measures of impulsivity. Appetite. (2015) 95:375–82. 10.1016/j.appet.2015.07.031 26235925

[62] Schag K Schönleber J Teufel M Zipfel S Giel KE. Food-related impulsivity in obesity and Binge Eating Disorder—a systematic review. Obes Rev. (2013) 14:477–95. 10.1111/obr.12017 23331770

[63] Loeber S Rustemeier M Paslakis G Pietrowsky R Müller A Herpertz S. Mood and restrained eating moderate food-associated response inhibition in obese individuals with binge eating disorder. Psychiatry Res. (2018) 264:346–53. 10.1016/j.psychres.2018.03.081 29674225

[64] Triggs WJ Cros D Macdonell RAL Chiappa KH Fang J Day BJ. Cortical and spinal motor excitability during the transcranial magnetic stimulation silent period in humans. Brain Res. (1993) 628:39–48. 10.1016/0006-8993(93)90935-G 8313168

[65] Matsugi A. Changes in the cortical silent period during force control. Somatosens Mot Res. (2019) 36:8–13. 10.1080/08990220.2018.1563536 30654690

[66] Lanza G Lanuzza B Aricò D Cantone M Cosentino FII Pennisi M. Direct comparison of cortical excitability to transcranial magnetic stimulation in obstructive sleep apnea syndrome and restless legs syndrome. Sleep Med. (2015) 16:138–42. 10.1016/j.sleep.2014.08.016 25534710

[67] Cantone M Bramanti A Lanza G Pennisi M Bramanti P Pennisi G. Cortical plasticity in depression. ASN Neuro. (2017) 9:175909141771151. 10.1177/1759091417711512 28629225 PMC5480639

